# Orphan Nuclear Receptor 4A1 (NR4A1) and NR4A2are Endogenous Regulators of CD71 and TheirLigands Induce Ferroptosis in Breast Cancer

**DOI:** 10.21203/rs.3.rs-6214709/v1

**Published:** 2025-04-21

**Authors:** Stephen Safe, Arafat Rahman Oany, Srijana Upadhyay, Wai Ning Tsui, Amanuel Hailemariam, Sarah Latka, John Landua, Sandra Scherer, Alana L Welm, Hugo Villanueva, Michael Lewis

**Affiliations:** Texas A&M University; Texas A&M University; Texas A&M University; Texas A&M University; Texas A&M University; Baylor College of Medicine; Baylor College of Medicine; University of Utah, Huntsman Cancer Institute and Department of Oncological Sciences; Department of Oncological Sciences, Huntsman Cancer Institute, University of Utah, Salt Lake City, UT 84112, USA; Baylor College of Medicine; Baylor College of Medicine

**Keywords:** NR4A1, NR4A2, Sp, Ferroptosis, Breast cancer

## Abstract

Ferroptosis is an iron-dependent cell death pathway that involves multiple genes including the transferrin receptor (TFRC/CD71), glutathione peroxidase 4 (GPX4) and SLC7A11. This study is based on the hypothesis that orphan nuclear receptor 4A1 (NR4A1) and NR4A2 maintain low levels of ferroptosis in triple negative breast cancer (TNBC) cells and that bis-indole derived (CDIM) compounds act as NR4A1/2 ligands that induce ferroptosis by enhancing CD71 expression. 1,1-Bis(3’-indolyl)-1-(3,5-disubstitutedphenyl)methane (DIM-3,5) analogs were investigated for their cytotoxicity and effects on NR4A1 and NR4A2 regulated genes and induction of ferroptosis by cytotoxicity, western blot and RT-PCR. Several assays also determined enhanced lipoperoxidation, reactive oxygen species and malondialdehyde formation in TNBC cells. Knockdown of NR4A1, NR4A2, Sp1 and Sp4 was carried out by RNA interference. Molecular mechanisms of NR4A1/2-mediated regulation of CD71 expression were determined using CD71-luciferase promoter constructs, overexpression of Sp1 and chromatin immunoprecipitation (ChIP) assays. Initial studies show that DIM-3,5 act as an inverse NR4A1/NR4A2 agonist that downregulated the pro-oncogenic responses/gene products regulated by both receptors in TNBC cells. DIM-3,5 analogs also induced ROS, malondialdehyde and lipoperoxide formation in TNBC cells and this was accompanied by indicators of ferroptosis that include decreased expression of GPX4 and SLC7A11 and induction of CD71. Induction of CD71, an important biomarker of ferroptosis was observed after treatment of TNBC cells with DIM-3,5 analogs, knockdown of NR4A1, NR4A2, Sp1 or Sp4 demonstrating that induction of CD71 was coregulated by both receptors. Moreover, both promoter and ChIP analysis indicated that NR4A1 and NR4A2 acted as ligand-dependent cofactors of Sp1/4-mediated expression of CD71 in TNBC cells. CD71, a key biomarker of ferroptosis is an NR4A1/2/Sp regulated gene that can be directly targeted by DIM-3,5 inverse NR4A1/2 agonists to induce ferroptosis in TNBC cells.

## INTRODUCTION

The orphan nuclear receptor 4A1 (NR4A) sub-family members NR4A1 (Nur77), NR4A2 (Nurr1) and NR4A3 (Nor1) are stress/inflammation-induced immediate-early genes that play key functional roles in maintaining cellular homeostasis and in pathophysiology [1, 2]. In inflammation and stress-induced diseases such as solid tumors NR4A1 is overexpressed and is a negative prognostic factor for lung, ovarian, colon and breast cancer patients [3–6]. NR4A2 is also highly expressed in solid tumors and is a negative prognostic factor for cervical, gastrointestinal, prostate, nasopharyngeal, colorectal, breast, gastric and pancreatic cancer patients [7–14]. Moreover, NR4A1 and NR4A2 knockdown result in decreased growth, migration and increased apoptosis in most solid tumors whereas the functional role and prognostic significance of NR4A3 is variable [15]. Studies in this laboratory have identified bis-indole derived compounds (CDIMs) that bind NR4A1 and NR4A2 and these include 1,1-bis(3’-indolyl)-1-(4-hydroxyphenyl)methane (DIM-4-OH) and 1,1-bis(3’-indolyl)-1-(4-chlorophenyl)methane (DIM-4-CI) respectively [16, 17]. Structure-activity studies have identified a series of 3,5-disubstitutedphenyl CDIM (DIM-3,5) analogs that inhibit mammary tumor growth in athymic nude mice bearing MDA-MB-231 cells at doses < 1 mg/kg/day [18] and the highly potent DIM-3,5 compounds have recently been identified as unique dual NR4A1/2 ligands that bind both NR4A1 and NR4A2, and act as inverse agonists in cancer cells [19]. Moreover, the DIM-3,5 ligands also decrease PD-L1 expression [20], and enhance CD8+/CD4 + T cell ratios in tumor infiltrating lymphocytes in a syngeneic orthotopic mouse model of breast cancer [18, 19].

Breast cancer is one of the most commonly diagnosed tumors in women and improved therapies coupled with increased awareness of this disease has resulted in a decrease of annual deaths from breast cancer [21]. However, treatment modalities for triple negative breast cancer (TNBC), a highly aggressive form of this disease have only had limited success [22]. Tumors from patients with TNBC do not express the estrogen receptor (ERα, ESR1), progesterone receptor (PR) or epidermal growth factor receptor 2 (ERBB2^+^) and the overall lack of prime molecular targets has negatively impacted identification of drugs for successfully treating TNBC. In recent years there has been increasing interest in development of ferroptosis-inducing agents for treating triple negative breast cancer; this type of lipoperoxide-induced cell death is dependent on multiple oxidative stress-induced pathways and obviates the need for targeting more well-established drug targets [23–25]. Several natural products with anticancer activities induce ferroptosis in triple negative breast cancer and other cell lines and these include quercetin, tetrandrine, flavonoids and other polyphenolics [26–29]. Recent studies show that quercetin, tetrandrine and flavonoids bind NR4A1 and inhibit NR4A1-dependent pro-oncogenic pathways and genes in breast and other cancer cell lines [30–33]. Based on our previous in vivo studies with DIM-3,5 dual NR4A1/2 ligands [18, 19], we hypothesize that NR4A1/2 may be important drug targets in TNBC due, in part, to their regulation of ferroptosis and key ferroptotic genes. This manuscript addresses the hypothesis and demonstrates for the first time that the transferrin receptor (CD71) is an NR4A1/2regulated gene that can be directly targeted by DIM-3,5 ligands to induce ferroptosis in TNBC.

## RESULTS

### Role of NR4A1 and NR4A2 as Pro-oncogenic Factors in TNBC

**Figure 1:**
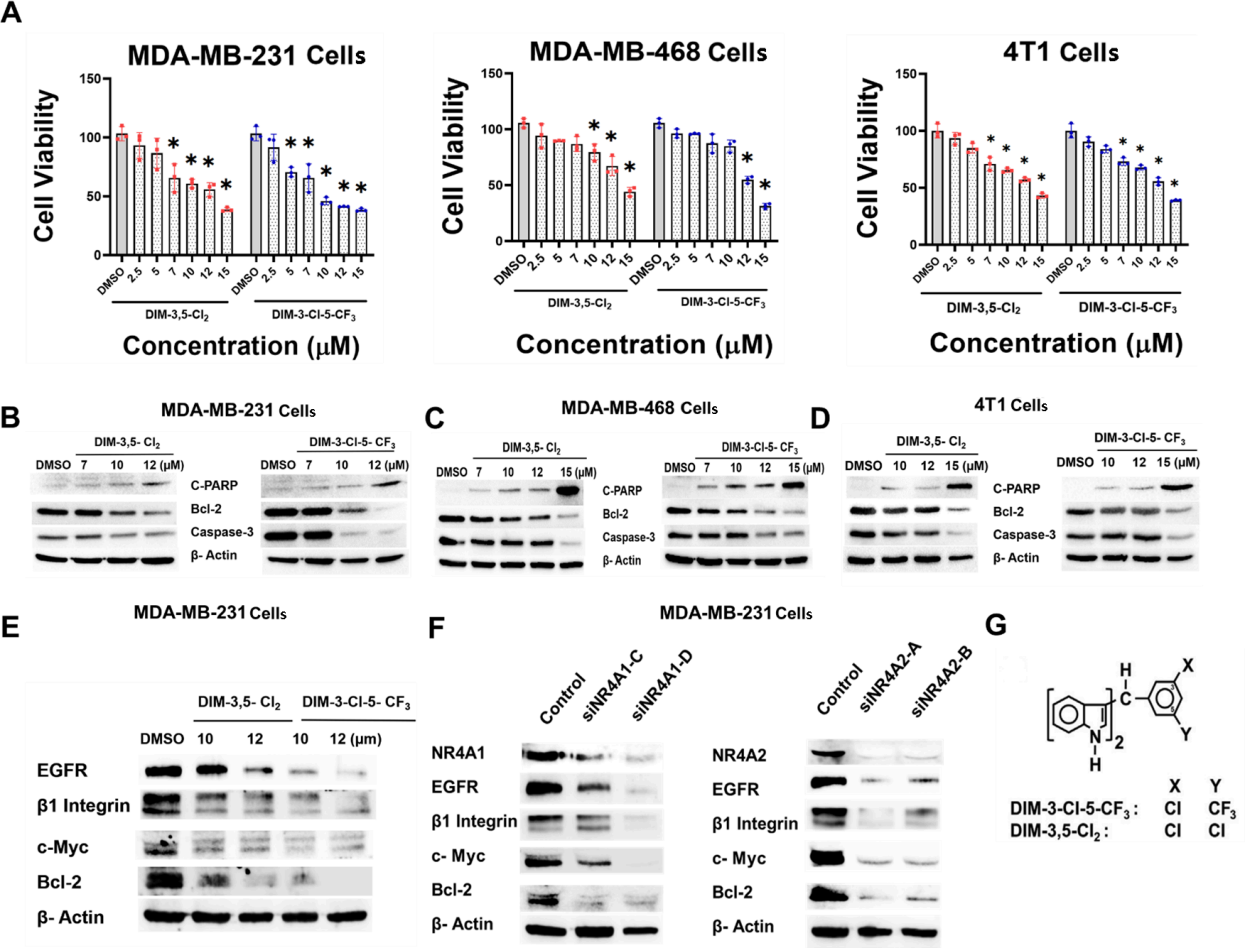
Effects of DIM-3,5 ligands and receptor knockdown on TNBC growth and selected gene products. **A**. MDA-MB-231, MDA-MB-468 and 4T1 cell were treated with DMSO and DIM-3,5 compounds and effects on cell viability were determined as outlined in the Methods. MDA-MB-231 (**B**), MDA-MB-468 (**C**) and 4T1 (**D**) cells were treated with DIM-3,5 compounds for 24 hours and whole cell lysates were analyzed by Western blots as outlined in the Methods. MDA-MB-231 cells were treated with DMSO and DIM-3,5 compounds (**E**) for 24 hours or transfected with oligonucleotides targeting NR4A1 (siNR4A1) or NR4A2 (siNR4A2) (**F**) and whole cell lysates were analyzed by Western blots as outlined in Methods. **G**. Structures of DIM-3,5 compounds. Cell viability studies were carried out in triplicate and results are expressed as means ± SD and significant (p < 0.05) inhibition is indicated (*).

Initial studies used DIM-3,5 analogs containing 3,5-dichlorophenyl (DIM-3,5-CI_2_) and 3-chloro-5-trifluoromethylphenyl (DIM-3-CI-5-CF_3_) groups and show that both compounds inhibited viability of human MDA-MB-231, MDA-MB-468 and mouse 4T1 TNBC cells (Fig. 1A). Treatment with DIM-3,5 compounds also induced cleaved PARP and caspase-3, and also decreased BCL-2 protein expression in MDA-MB-231, MDA-MB-468 and 4T1 (Figs. 1B–1D) demonstrating the proapoptotic activity of DIM-3,5 compounds that bind both receptors and act as inverse agonists [15, 18]. Previous studies have characterized several NR4A1-regulated genes including *EGFR, β1-integrin, c-Myc and bcl-2* that are downregulated after NR4A1 knockdown or treatment with CDIM compounds [15]. Using MDA-MB-231 cells as a model, both DIM-3,5-CI_2_ and DIM-3-CI-5-CF_3_ decreased expression of these gene products (Fig. 1E), and similar results were observed after knockdown of NR4A1 or NR4A2 by RNA interference (Fig. 1F). Thus like NR4A1, NR4A2 also regulates expression of a similar set of pro-oncogenic gene products, and this is consistent with the downregulation of these proteins by DIM-3,5 dual NR4A1/2 inverse agonists.

### Dual NR4A1/2 Ligands Induce Ferroptosis in TNBC Cells

**Figure 2:**
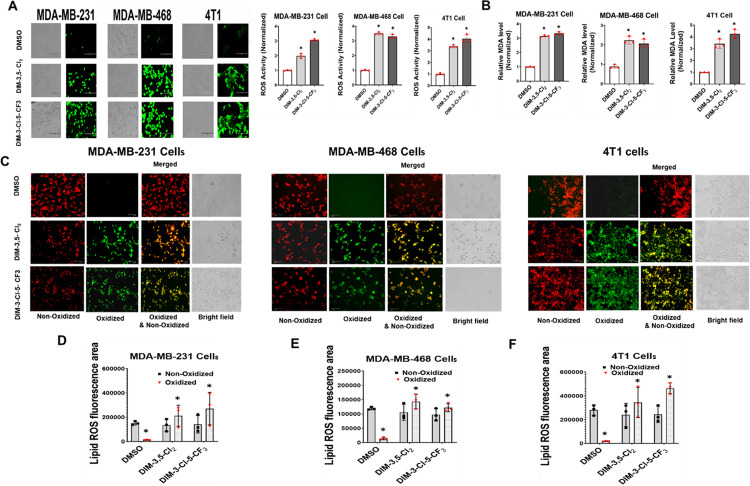
Induction of ROS induced lipoperoxidation by DIM-3,5 compounds. TNBC cells were treated with DMSO and DIM-3,5 compounds at a concentration of 12 μM/L for 16 hours and ROS (**A**) and MDA (**B**) and lipoperoxidation (**C**) were determined and quantitated fluorometrically as outlined in the Methods. The quantitation of lipoperoxidation using BIODIPYä 581/591 C11 staining is outlined as MDA-MB-231 (**D**), MDA-MB-468 (**E**), and 4T1 (**F**) cells. Results (**A-F**) are means ± SD of 3 replicate treatments and significant (p < 0.05) induction of ROS, MDA formation, and lipoperoxides formation (oxidized control vs treatment) are indicated (*).

Ferroptosis is dependent on induction of ROS which is required for Fe^3+^-dependent production of lipoperoxides that result in fatty acid degradation and cell damage. Results in Fig. 2A show that DIM-3,5-CI_2_ and DIM-3-CI-5-CF_3_ significantly induce ROS in MDA-MB-231, MDA-MB-468 and 4T1 cells (16-hour treatment) as determined using the cell permeable H_2_CDCFDA dye. Lipoperoxidation breakdown induces significant cell damage and fatty acid degradation resulting in formation of malondialdehyde (MDA) and DIM-3,5-CI_2_ and DIM-3-CI-5-CF_3_ significantly induced MDA formation in the 3 TNBC cell lines (Fig. 2B). The subsequent formation of oxidized lipids is a hallmark of ferroptosis [34, 35] and results summarized in Figs. 2C show that DIM-3,5-CI_2_ and DIM-3-CI-5-CF_3_ induce lipoperoxidation in MDA-MB-231, MDA-MB-468 and 4T1 cells using BODIPY^™^ 581/591 C11 as a sensor of lipoperoxide formation. Quantitation of the results showed that DIM-3,5 ligands enhanced formation of lipoperoxides (oxidized) (Fig. 2D–2F). These results clearly demonstrate that the dual NR4A1/2 ligands induce ferroptosis as evidenced by induction of these diagnostic oxidative pathways and therefore, we further investigated the role of NR4A1/NR4A2 and the DIM-3,5 analogs on modulation of key ferroptotic genes.

**Figure 3:**
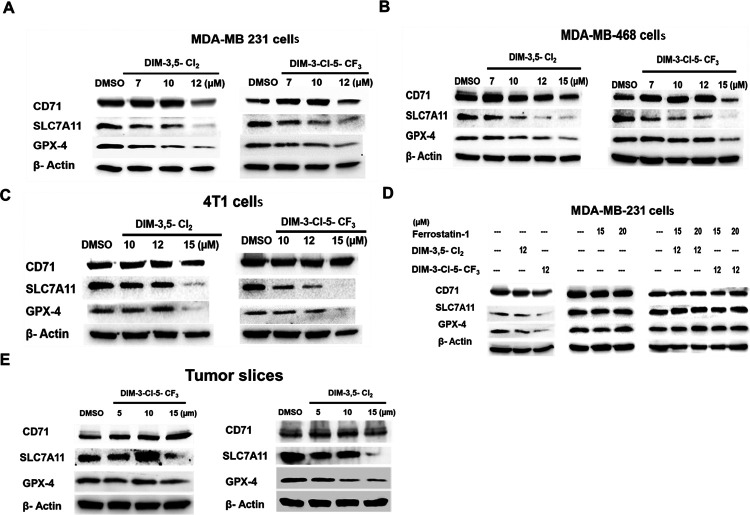
DIM-3,5 compounds modulate expression of ferroptotic gene products in TNBC cells. MDA-MB-231 (**A**), MDA-MB-468 (**B**) and 4T1 (**C**) cells were treated with DMSO or DIM-3,5 compounds for 24 hours and whole cell lysates were analyzed by Western blots to determine changes in key ferroptotic genes. **D**. Effect of ferrostatin-1 on DIM-3,5 compounds alone and in combination were determined in MDA-MB-231 cells after treatment for 24 hours, and whole cell lysates were analyzed by Western blots as outlined in the Methods. (**E**) Tumor slices were obtained as outlined in the Methods and after treatment with DIM-3,5 analogs for 72 hours whole cell lysates were obtained and analyzed by western blots as outlined in the Methods.

Treatment of TNBC cells with DIM-3,5-CI_2_ and DIM-3-CI-5-CF_3_ for 24 hours, decreased SLC7A11 and GPX4 proteins in MDA-MB-231, MDA-MB-468 and 4T1 cells (Figs. 3A – 3C, respectively). The concentration-dependent effects of DIM-3,5 compounds on CD71 expression were somewhat variable in the 3 breast cancer cell lines. In 4T1 cells, CD71 protein levels were not decreased after treatment with 10–15 μmol/L DIM-3,5 compounds for 24 hours whereas in MDA-MB-231 and MDA-MB-468 cells, 7 and 10 μmol/L DIM-3,5 analogs induced levels of CD71 protein at one or both concentrations but 12 or 15 μmol/L DIM-3,5 downregulated CD71 protein expression. Figure 3D summarizes the effects of the ferroptosis inhibitor ferrostatin1 on CD71, SLC7A11 and GPX4 expression alone or in combination with 12 μmol/L DIM-3,5 compounds. DIM-3,5 compounds alone decreased expression of CD71, SLC7A11 and GPX4 proteins at the high 12 μmol/L concentration whereas 15 and 20 μmol/L ferrostatin-1 alone did not affect levels of these proteins in MDA-MB-231 cells. In combination studies, ferrostatin-1 inhibited the DIM-3,5-mediated downregulation of all 3 proteins thus inhibiting the pro-ferroptotic effects of DIM-3,5 analogs on downregulation of GPX4 and SLC7A11. Similar results were also observed in tumor slices treated with the DIM-3,5 analogs (Fig. 3E).

### DIM-3,5 Dual NR4A1/2 Ligands Induce CD71 Expression in TNBC Cells

**Figure 4:**
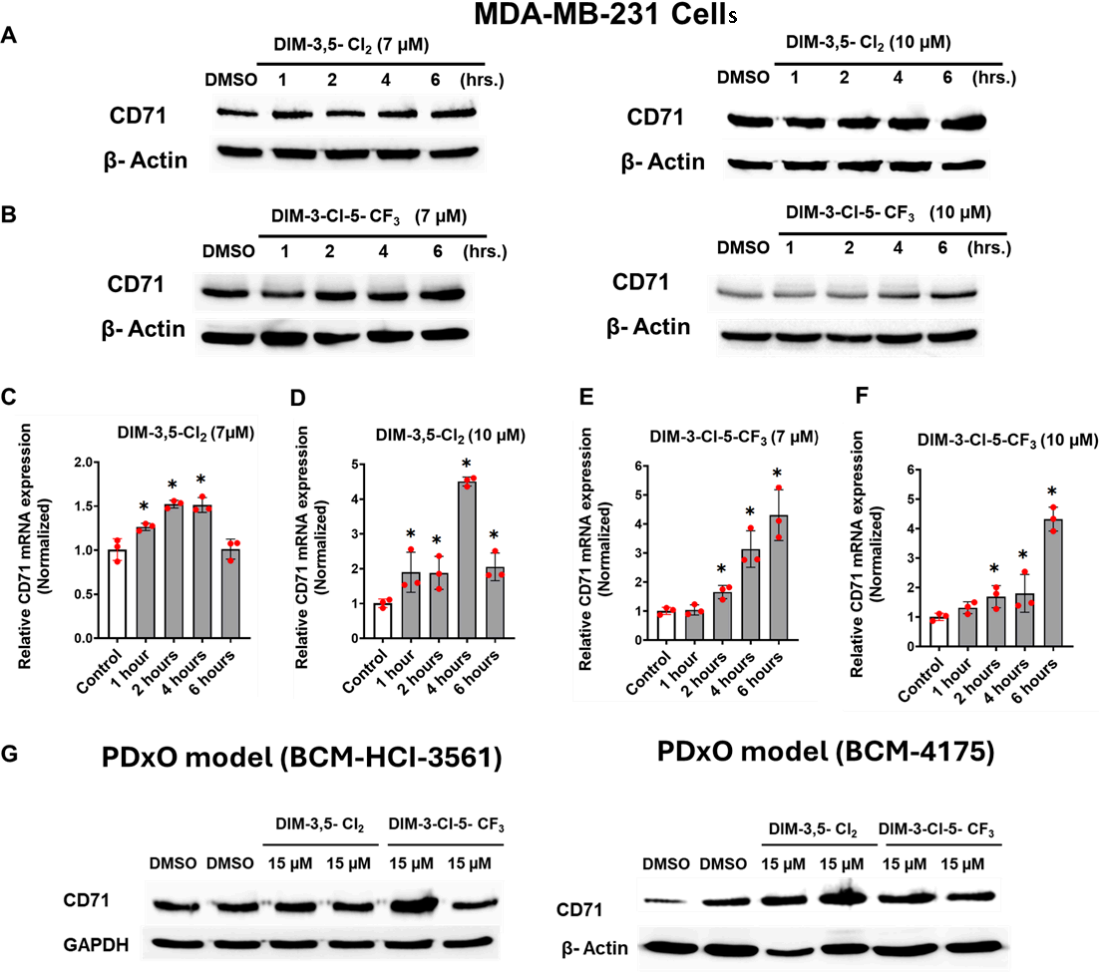
Induction of CD71 by DIM-3,5 compounds. MDA-MB-231 cells were treated with DMSO, 7 and 10 μM DIM-3,5-CI_2_ (**A**) and 7 and 10 μM DIM-3-CI-5-CF_3_ (**B**) for up to 6 hours and whole cell lysates were analyzed by Western blots as outlined in the Methods. **C**. PDxO organoids were treated with 15 μM DIM-3,5 compounds for 24 hours and whole cell lysates were analyzed for CD71 expression by Western blots as outlined in the Methods. The time dependent (0–6 hours) induction of CD71 mRNA by 7 and 10 μM DIM-3,5-CI_2_ (**D** and **E**) and 7 and 10 μM DIM-3-CI-5-CF_3_ (**F** and **G**) was determined by RT-PCR as outlined in the Methods. Results (**D-G**) are means ± SD for replicate (3) determinations and significant (p < 0.05) induction is indicated (*).

Overexpression of CD71 is a confirmed biomarker of ferroptosis [36] and results illustrated in Fig. 4 showed some variability with respect to the effects of DIM-3,5 analogs on levels of CD71 protein; downregulation was primarily observed at high concentrations of DIM-3,5 analogs after treatment for 24 hours whereas induction of CD71 was observed at lower concentrations. Therefore, we further investigated the time- and DIM-3,5 concentration-dependent effects of this gene in MDA-MB-231 cells. DIM-3,5-CI_2_ (7 and 10 μmol/L) induced CD71 protein levels over a 1–6-hour period (Fig. 4A) and similar results were observed for DIM-3-CI-5-CF_3_ (Fig. 4B) however the pattern of induction of CD71 protein was both compound, concentration and time-dependent. A similar approach was used for determining the time-dependent induction of CD71 mRNA levels in MDA-MB-231 (Figs. 4C–4F). Both 7 and 10 μmol/L DIM-3,5-CI_2_ and DIM-3-CI-5-CF_3_ induced CD71 mRNA levels however, the time courses of the maximal induction responses were variable and compound-dependent. To investigate effects of DIM-3,5-CI_2_ and DIM-3-CI-5-CF_3_ in pre-clinical 3D models of TNBC, we tested these compounds using NR4A1/2expressing PDX-derived organoid (PDxO) models and observed induction of CD71 after treatment with 15 μmol/L concentrations of these dual receptor ligands (Fig. 4G). These results suggest that CD71 expression is induced by the dual NR4A1/2 ligands acting as agonists.

### DIM-3,5 Analogs Activate NR4A1/NR4A2:Sp to Induce CD71 in TNBC cells

**Figure 5:**
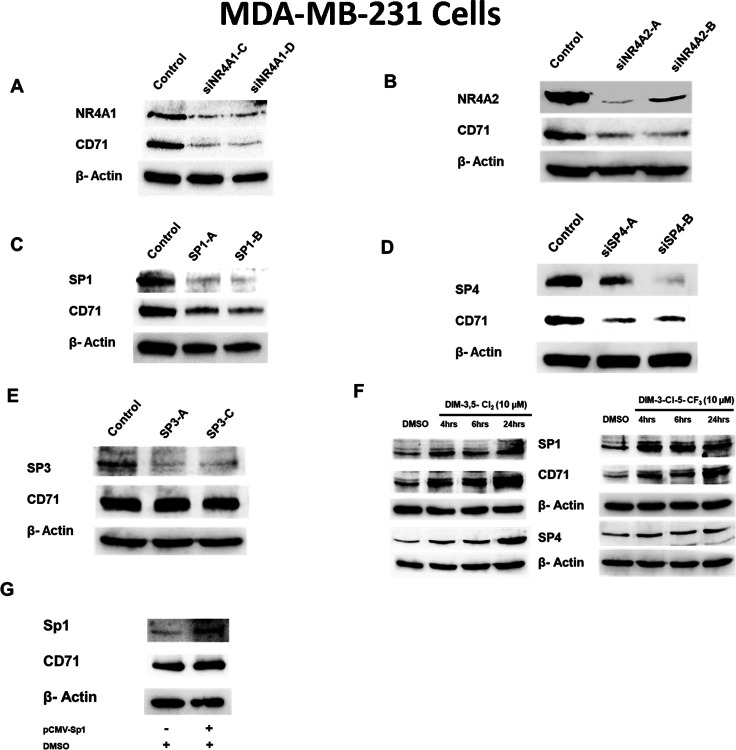
Role of NR4A1 and Sp TFs in the regulation of CD71. MDA-MB-231 cells were transfected with oligonucleotides targeting NR4A1 (**A**), NR4A2 (**B**), Sp1 (**C**), Sp4 (**D**) and Sp3 (**E**) and after 72 hours, whole cell lysates were analyzed by Western blots as outlined in the Methods. (**F**) Cells were treated with 10 μM DIM-3,5-CI_2_ and DIM-3-CI-5-CF_3_ for up to 24 hours and effects on CD71, Sp1 and Sp4 were determined by Western blots of whole cell lysates as outlined in the Methods. (**G**) Effects of Sp1 overexpression on CD71 were obtained by transfection of the p^CVM-Sp1^ expression plasmid and subsequent western blot analysis of whole cell lysates as outlined in the Methods.

Results illustrated in Fig. 5A and 5B show that knockdown of NR4A1 and NR4A2 by RNA interference decreased expression of CD71 in MDA-MB-231 cells demonstrating that both receptors coregulate expression of this key ferroptotic gene. It has previously been reported that Sp1 plays an important role in regulating expression of CD71 through interactions with GC-rich sites in the CD71 gene promoter [37]. Previous studies also show that NR4A1 and other nuclear receptors can act as nuclear cofactors that interact with DNA bound Sp1 to regulate basal gene expression in cancer cells and receptor ligands such as DIM-3,5 compounds can modulate (inhibit or induce) expression of the Sp-regulated genes through a cofactor-dependent pathway [38]. Figures 5C–5D illustrate that knockdown of Sp1 and Sp4, but not Sp3 (Fig. 5E), decrease expression of CD71 in MDA-MB-231 cells suggesting that both NR4A1 and NR4A2 cooperatively act as nuclear cofactors to activate Sp1- and Sp4-dependent *CD71* expression. This complements results of previous studies showing that NR4A1 coactivated Sp1 or Sp4, but not Sp3, and in these studies the CDIM-derived NR4A ligands act as inverse agonists to inhibit NR4A1/Sp1:Sp4-mediated expression of PD-L1, PAX3-FOX01, G9a, β1- and several other integrins in cancer cell lines [20, 39–43]. In contrast, the DIM-3,5 dual NR4A1/2 ligands act as receptor agonists for induction of CD71. Results illustrated in Fig. 5F confirm that in MDA-MB-231 cells treated with 10 μmol/L DIM-3,5 expression of Sp1 and Sp4 is somewhat increased, and this is consistent with the enhanced expression of CD71. In addition, the overexpression of the Sp1 (pCMV-Sp1) plasmid enhances CD71 protein expression and this supports the involvement of both NR4A1/2 and Sp1 in regulating CD71 (Fig. 5G).

**Figure 6:**
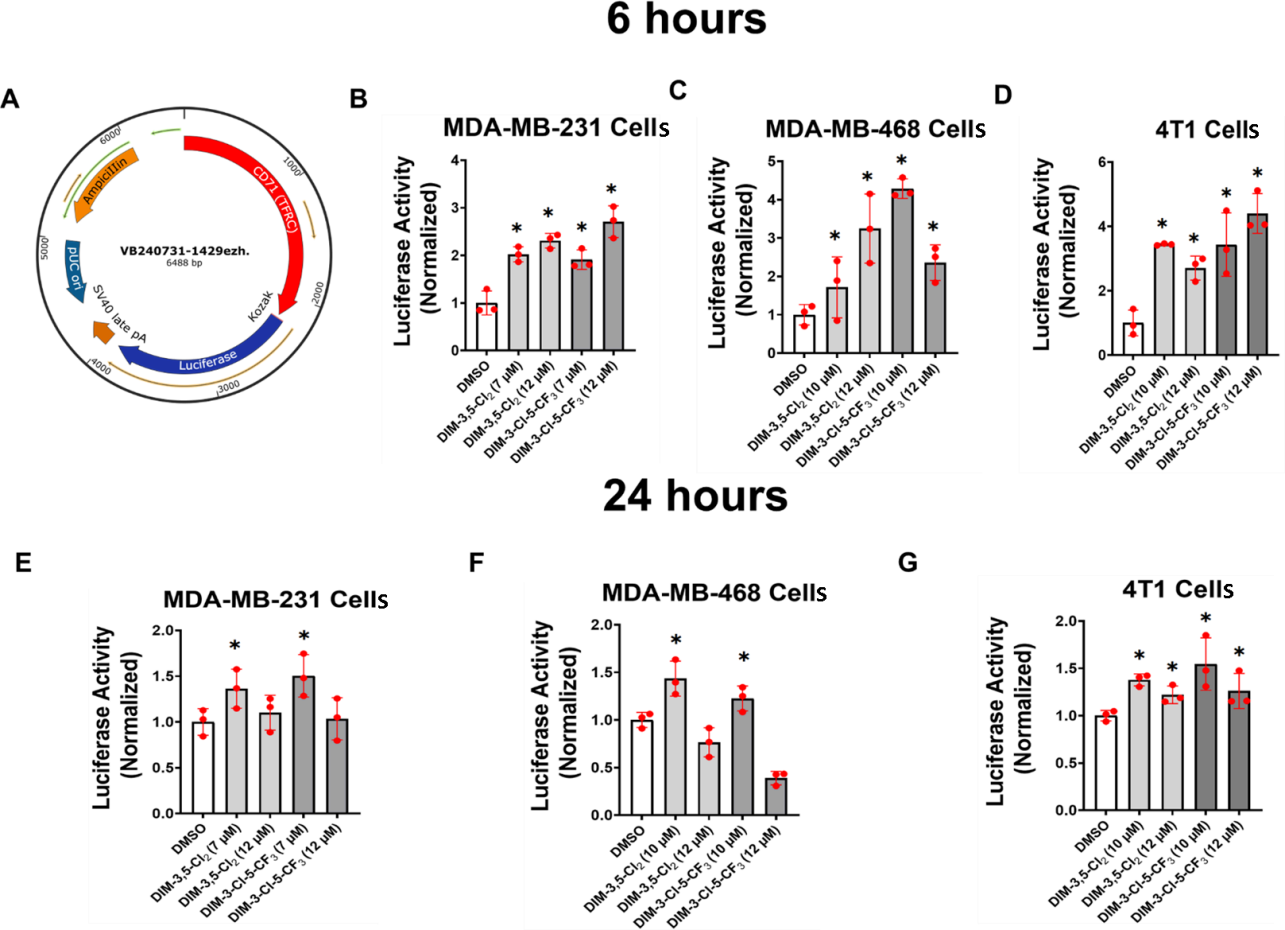
Induction of CD71-luc activity by DIM-3,5 compounds. (**A**) The vector construct is outlined and −2000 to +200 region of the CD71 promoter was cloned into a Mammalian Promoter-Testing Vector (pRP [Pro]-{CD71}>Luciferase). .MDA-MB-231 (**B**). MDA-MB-468 (**C**) and 4T1 (**D**) cells were transfected with CD71-luc construct and treated with DMSO and DIM-3,5 ligands (7–12 μM) for **6 hours** and luciferase activity was determined as outlined in the Methods. MDA-MB-231 (**E**), MDA-MB-468 (**F**) and 4T1 (**G**) cells were transfected with CD71-luc construct and treated with DMSO and DIM-3,5 compounds (7–12 μM) for 24 hours and luciferase activity was analyzed as outlined in the Methods. Results are expressed as means ± SD for replicate (3) determinations for each treatment group and significant (p < 0.05) induction (compared to DMSO controls) is indicated (*).

**Figure 7:**
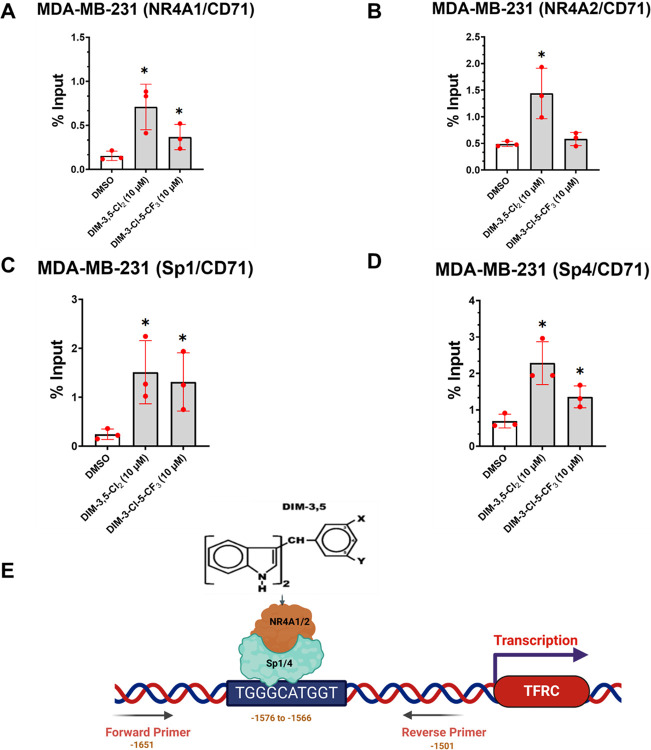
Chromatin immunoprecipitation results in MDA-MB-231 cells. Interactions of NR4A1 (**A**), NR4A2 (**B**), Sp1 (**C**) and Sp4 (**D**) with the CD71 gene promoter containing the Sp binding region was determined after treatment with DIM-3,5 compounds in a ChIP assay (in triplicate) as outlined in the Methods section. (**E**) Summary of the CD71 gene promoter, Sp binding sites (−1576 to - 1566) and the primers used for detecting protein interactions in this region of the promoter. Results are expressed as means ± SD for replicate (3) determinations for each treatment group (**A-D**) and significant (p < 0.05) induction is indicated (*).

Therefore, we further investigated the induction of CD71 by DIM-3,5 compounds using a CD71-luc construct containing the Sp binding elements − 1576 to −1566 of the CD71 promoter (Fig. 6A) that have previously been identified as an important region for regulating CD71 expression [37]. In MDA-MB-231 cells transfected with CD71-luc [pRP [Pro]-{CD71}>Luciferase] construct for 6 hours we observed that DIM-3,5 compounds significantly induced luciferase activity (Fig. 6B–6D) in MDA-MB-231, MDA-MB-468 and 4T1 cells which is consistent with previous studies showing that transactivation of CD71 was primarily associated with this region of the promoter [37]. In TNBC cells transfected with the CD71-luc construct treatment with DIM-3,5 analogs for 24 hours also induced luciferase activity (Fig. 6E–6G) but in some cells the high concentration decreased luciferase activity. These results complemented the effects of DIM-3,5 analogs on CD71 mRNA and protein levels in TNBC cells. Figure 6A summarizes the structure of the CD71-luc construct. The associations of the putative nuclear factors NR4A1, NR4A2, Sp1 and Sp4 that regulate expression of CD71 with the CD71 gene promoter were investigated in a ChIP assay using primers that encompass the “TGGGCATGGT” active region of the CD71 gene promoter [41]. In solvent (DMSO/control) treated MDA-MB-231 cells subsequent analysis by RT-PCR showed that NR4A1, NR4A2, Sp1 and Sp4 were associated with the promoter (Figs. 7A–7D). Treatment of MDA-MB-231 with either DIM-3,5-CI_2_ or DIM-3-CI-5-CF_3_ for 6 hours either did not affect or increased association of NR4A1, NR4A2, Sp1 and Sp4 with the CD71 promoter. The enhanced ligand-dependent interactions of NR4A and Sp with CD71 promoter correlated with the induction of CD71 by DIM-3,5 analogs. In contrast, for genes such as PD-L1 that are repressed by DIM-3,5 compounds in TNBC cells [20] there is a decrease of one or more of NR4A1 and Sp bound to the GC-rich PD-L1 promoter, and this is observed in other genes downregulated by NR4A/Sp [38–43]. A proposed model for the induction of CD71 expression by NR4A1 and NR4A2 is depicted in Fig. 7E, and this pathway allows for the direct targeting of this key pro-ferroptotic gene.

## DISCUSSION

Studies in this laboratory have shown the CDIM compounds that act as functional NR4A1 inverse agonists in cancer cells inhibit TNBC cell and tumor growth and the EC_50_ values for inhibiting tumor growth in athymic nude mice bearing MDA-MB-231 cells by DIM-3,5 analogs is < 1 mg/kg/day [18]. This dose was much lower than observed for some initial DIM compounds that contained only a 4-hydroxyphenyl moiety [DIM-4-OH]. The potent antitumorigenic activity of the DIM-3,5 analogs are due to several factors including their activity as dual NR4A1/NR4A2 inverse agonists that inhibit the pro-oncogenic pathway/genes, such as PD-L1, regulated by both receptors in cancer cells [18, 19]. DIM-3,5 analogs also enhance immune surveillance and reverse CD8^+^ and CD4^+^ T cell exhaustion in a syngeneic mouse model of colon cancer [43]. We hypothesized that DIM-3,5 NR4A1/2 ligands also target other receptor-dependent pathways in TNBC and that one of these may be the induction of ferroptosis, an ROS-dependent cell death pathway. This hypothesis was based on previous reports showing that several natural products including polyphenolic such as quercetin, tetrandrine and resveratrol all induce ferroptosis in cancer cells [24–29] and studies in this laboratory show that these compounds also bind NR4A1 and exhibit inverse NR4A1 agonist activity [30–33] similar to that observed for DIM-3,5 analogs [18]. In addition, the NR4A1 ligand DIM-4-OH downregulates expression of stearoyl-CoA-desaturase in pancreatic cancer cells to activate ferroptosis [44] however, in this study expression of the desaturase was minimal in TNBC cells.

DIM-3,5-CI_2_ and DIM-3-CI-5-CF_3_ were recently characterized as ligands that bind both NR4A1 and NR4A2 and inhibit NR4A1/NR4A2-dependent genes and pathways in cancer cells [18, 19, 47] and this was confirmed in TNBC cells as illustrated in Fig. 1. Both compounds inhibited breast cancer cell growth and induced apoptosis and there was a concordance between the effects of NR4A1 and NR4A2 knockdown and effects of DIM-3,5 analogs in MDA-MB-231 cells and on several NR4A-regulated genes including *EGFR, β1-integrin, bcl-2* and *c-Myc*. Quercetin which is an NR4A1 ligand induces many of these same responses in Rh30 rhabdomyosarcoma cells [32] and also induces ferroptosis in breast and other cancer cell lines [26, 28]. The parallel functions between DIM-3,5 analogs and quercetin can also be extended to their effects on ferroptosis since DIM-3,5-CI_2_ and DIM-3-CI-5-CF_3_ induce ROS, lipoperoxidation and MDA formation and also decrease expression of GPX4 and SLC7A11 in TNBC cell lines and similar results have been observed for flavonoids in cancer cells [25–29]. CD71 also plays a key role in ferroptosis by facilitating the transport of Fe^3+^ into the cell and the combination of Fe^3+^ ROS can lead to lipoperoxidation [45, 46].

CD71 expression has been reported as an important biomarker of ferroptosis [40] and has a primary function of facilitating Fe^3+^ uptake into the cell. DIM-3,5 compounds induce CD71 gene and gene product formation in MDA-MB-231 and other TNBC cells. However, this response is both concentration and time-dependent and high concentrations tend to decrease levels of CD71. Sp1 and Sp4 knockdown resulted in decreased CD71 levels, and this is consistent with previous reports showing that Sp1 interactions with the − 1576 to −1566 region of the CD71 gene promoter are important for CD71 expression [37]. However, NR4A1 and NR4A2 knockdown also decreased levels of CD71 protein demonstrating the NR4A1 and NR4A2 cooperatively regulated CD71 expression by acting as cofactors where Sp1 and Sp4 transcription factors bind to GC-rich gene promoter elements. Thus, NR4A1 and NR4A2 act as ligand-dependent cofactors of Sp1/4 and this is consistent with results of ChIP assays showing that Sp1/4 and NR4A1/NR4A2 interact with the transcriptionally active GC-rich region of the CD71 promoter and their expression is either unchanged or induced after treatment with DIM-3,5 compounds. Nuclear receptors such as NR4A1 and NR4A2 interact with multiple proteins including DNA bound transcription factors such as Sp and AP-1 to regulate endogenous expression of multiple genes [38]. For example, ERα/Sp regulates expression of cyclin D1 through interaction with GC-rich sites and ERα agonists and antagonist can induce or repress cyclin D1 expression respectively [38, 47]. Knockdown of NR4A1, NR4A2 or Sp1/4 also decrease expression of NR4A1/Sp-regulated genes such as PD-L1, β1- and other integrins in breast cancer cells [20, 40, 42] and similar results were observed for CD71 in this study demonstrating that NR4A1 and NR4A2 in combination with Sp1 and Sp4 regulate basal expression of this key ferroptotic gene. Previous studies have identified NR4A1/Sp1-regulated genes such as PD-L1 and β1-integrin in TNBC cells [20, 42] that are downregulated after treatment with DIM-3,5 ligands which act as inverse agonists. These effects are usually accompanied by decreased association of NR4A1, NR4A2 or Sp1/4 with the target gene promoter as determined in ChIP assays. In contrast, the induction of CD71 by DIM-3,5 compounds increase association of one or more of NR4A1, NR4A2, Sp1 or Sp4 with the transcriptionally active GC-rich region of the CD71 promoter. In summary, these results further define the mechanism of CD71 expression and induction in TNBC cells and demonstrate that NR4A1 and NR4A2 coregulate not only endogenous expression of CD71 but also that dual NR4A1/NR4A2 ligands represent a novel class of mechanism-based inducers of CD71 and ferroptosis. Future studies will investigate ferroptosis-induced damage associated molecular patterns (DAMPs) by DIM-3,5 analogs and their direct effects on immune cells and also their interactions with NR4A1/2 ligand-mediated immune responses.

## MATERIALS AND METHODS

### Cell culture, reagents, ligands and cell proliferation

The breast cancer cell lines MDA-MB-231(CRM-HTB-26) and MDA-MB-468 (HTB-132) from humans and mouse 4T1 (CRL-2539) cancer cells were purchased from American Type Culture Collection (Manassas) and routinely checked for mycoplasma contamination. The maintenance and growth of these cell lines and effects of compound on cell proliferation was carried out as previously described [18, 20, 42].

### Tissue slice and PDxO experiments

The Baylor College of Medicine (BCM) Institutional Animal Care and Use Committee (IACUC) approved all procedures using live animals. Female immune-compromised mice (SCID/beige, Inotiv stock 186) were used to grow the previously established patient-derived xenograft (PDX) model, BCM-15120. Protocols and surgical procedures used to generate BCM-15120 have been described [48, 49]. Tumor discs from PDX were immediately rinsed in sterile PBS and transferred to a 24-well gas permeable tissue culture plate (Coy Laboratory) containing media [50] treated with DIM-3,5-Cl_2_; media and compounds were replaced every 24 hours and after 72 hours, whole cell lysates of the tumor discs were obtained and analyzed by Western blots. Tumor organoids derived from patient-derived xenografts (PDX) were established as described [50, 51], treated with DIM-3,5-Cl_2_ and DIM-3-Cl-5-CF_3_ for 24 hours and whole cell lysates were analyzed by Western blots [18–20].

### Measurement of ROS

The cell permeable dye-CM-H_2_DCFDA (Invitrogen, # C6827) was used as an indicator for the detection of reactive oxygen species (ROS) in cells. Human (MDA-MB-231 and MDA-MB-468) and mouse (4T1) breast cancer cells were cultured at a density of 3.0 × 10^5^ cells per well for 24 hours, treated with 12 μmol/L DIM-3,5-Cl_2_, DIM-3-Cl-5-CF_3_ and DMSO for 16 hours, and ROS was determined according to the manufacturer’s protocol and as described [52].

### Lipid peroxidation and malondialdehyde (MDA) assays

The BODIPY^™^ 581/591 C11 (Life Technologies, # D3861) was used for determination of lipoperoxidation. The fluorescence properties of this probe shifted from red signals (581/610 nm) to green signals (484/510 nm) upon oxidation of the polyunsaturated butadienyl segment of fatty acids in live cells. Breast cancer cells were treated with DMSO 12 μmol/L, DIM-3,5-Cl_2_, and DIM-3-Cl-5-CF_3_ for 16 hours and using the BIODIPY^™^ probe cells were analyzed for lipoperoxidation according to the manufacturer’s protocol. MDA was measured and quantitated using the Lipid Peroxidation (MDA) Assay Kit (Sigma-Aldrich, # MAK568) following the manufacturer’s protocol using 12 μmol/L CDIM compounds and treatment time of 16 hours. The MDA content was calculated for individual treatment groups from the standard curve.

### Western blotting and Quantitative real-time polymerase chain reaction (qPCR) assay

Cells were treated with vehicle (DMSO) and ligands (DIM-3,5 compounds and Ferrostatin-1), whole cell lysates were analyzed by Western blots as described [18–20]. Antibodies used for the protein detection are listed in the **Supplementary Table 1**. For determining CD71 (TFRC) mRNA, cells were treated with DIM-3,5 compounds, total RNA content was extracted using the RNeasy Mini Kit (QIAGEN, # 74104) and qPCR was performed using the amfiSure qGreen Q-PCR Master Mix (GenDEPOT) as described [53]. The β-Actin primer sequences forward: 5’-ATCGGTTGGTGCCACTGAATGG-3’ and reverse: 5’-AGGTCTTTGCGGATGTCCACGT-3’ were used as a reference gene to normalize the input cDNA and the primer sets for the CD71 were forward: 5’- ATCGGTTGGTGCCACTGAATGG-3’ and reverse 5’-ACAACAGTGGGCTGGCAGAAAC-3’.

### Small RNA interference (siRNA) and plasmid transfection and luciferase assays

Cells were seeded at a density of 1.5 × 10^5^ cells per well on a 6-well plate. Lipofectamine RNAiMAX (Invitrogen, # 56531) was used for the transfection and carried out according to the manufacturer’s protocol with the mixture of Gibco-Opti-MEM (Fisher Scientific) and oligos. After 72 hours, cells were harvested and lysed with RIPA buffer for protein analysis. The siRNAs that were used for this study are siNR4A1 (SASI_Mm01_00077215-NR4A1_C, SASI_Mm01_00077216-NR4A1_D), siNR4A2 (SASI_Hs02_0034_1056-NR4A2-A, SASI_Hs02_0034_1057-NR4A2-B), siSp1 (SASI_Hs02_00333289-Sp1-A, SASI_Hs02_00070994-Sp1-B), siSp3 (SASI_Hs01_00211941-Sp3-A, SASI_Hs01_00211941=-Sp3-C), siSp4 (SASI_HS01–00114420-Sp4-A, SASI_HS01–00114421-Sp4-B) and Scrambled siRNA (CGU ACG CGG AAU ACU UCG A (Sigma-Aldrich). The plasmid containing Sp1 coding sequence (Addgene plasmid # 12097) was transfected using Lipofectamine^™^ 3000 reagent (Invitrogen, # L3000008) according to the manufacturer’s instructions. After 48 hours, cells were harvested and lysed with RIPA buffer for protein analysis by Western blot. The CD71 (TFRC) promoter construct CD71-(luc) contained a −2000 to + 200 CD71 promoter insert that was purchased from VectorBuilder (VectorBuilder Inc., # VB240731–1429ezh). The experiment was carried out following the manufacturer’s protocol and as previously described [53].

### Chromatin immunoprecipitation (ChIP) assay

The ChIP assay was conducted using the ChIP-IT Express kit (Active Motif, # 53008), following the manufacturer’s protocol and primer sets for this gene (**Supplementary Table 2**). Cells were treated with DIM-3,5 compounds at a concentration of 10 μmol/L, along with vehicle control (DMSO), and binding to the CD71 promoter using qPCR was determined as described [53].

## STATISTICAL ANALYSIS

Statistical analysis was performed using a t test. All the in vitro experiments were carried out in triplicate. Data are expressed as the mean ± SD. One-way analysis of variance (Dunnett’s) was used to determine statistical significance, and P values < 0.05 were considered statistically significant.

## Funding

Syd Kyle endowment; NIH sponsored: P30 Environmental Center (ESO29067), RO1 grant (CA289580), P30 Cancer Center Support Grant (NCI-CA125123) and U54 Cooperative Agreement (U54CA224076); CPRIT Core Facility Award (RP220646).

## Data Availability

The datasets used and/or analyzed during the current study are available from the corresponding author on reasonable request.

## Abbreviations

BCM: Baylor College of Medicine
CDIM: bis indole derived
ChIP: chromatin immunoprecipitation
DIM: 1,1-bis(3’-indolyl)methane
DMSO: dimethylsulfoxide
EGFR: epidermal growth factor receptor
ERα: estrogen receptorα
GPX4: glutathione peroxidase4
NR4A: nuclear receptor
PBS: phosphate-buffered saline
PCR: polymerase chain reaction
PDXO: patient derived xenograft organoids
ROS: reactive oxygen species
TBA: thiobarbituric acid
TNBC: triple negative breast cancer
